# Circulating cell-free DNA methylation patterns as non-invasive biomarkers to monitor colorectal cancer treatment efficacy without referencing primary site mutation profiles

**DOI:** 10.1186/s12943-023-01910-y

**Published:** 2024-01-03

**Authors:** Kazuya Yasui, Toshiaki Toshima, Ryo Inada, Yuzo Umeda, Shuya Yano, Hiroaki Tanioka, Akihiro Nyuya, Toshiyoshi Fujiwara, Takeshi Yamada, Yoshio Naomoto, Ajay Goel, Takeshi Nagasaka

**Affiliations:** 1 Department of Gastroenterological Surgery, Dentistry and Pharmaceutical Sciences, Okayama University Graduate School of Medicine, Okayama, 700-8558 Japan; 2 Department of Gastroenterological Surgery, Kochi Health Sciences Centre, Kochi, 781-0111 Japan; 3 Department of Clinical Oncology, Kawasaki Medical School, 577 Matsushim, Kurashiki, 701-0912 Japan; 4 Department of Gastrointestinal and Hepato-Biliary-Pancreatic Surgery, Nippon Medical School, Tokyo, 113-8602 Japan; 5 Department of General Surgery, Kawasaki Medical School, Okayama, 700-8505 Japan; 6 Department of Molecular Diagnostics and Experimental Therapeutics, Beckman Research Institute of City of Hope, Biomedical Research Center, Monrovia, CA 91016 USA

**Keywords:** Circulating cell-free DNA, Liquid biopsy, Methylation, Colorectal cancer

## Abstract

This study investigates methylation patterns in circulating cell-free DNA (ccfDNA) for their potential role in colorectal cancer (CRC) detection and the monitoring of treatment response. Through methylation microarrays and quantitative PCR assays, we analyzed 440 samples from The Cancer Genome Atlas (TCGA) and an additional 949 CRC samples. We detected partial or extensive methylation in over 85% of cases within three biomarkers: *EFEMP1*, *SFRP2*, and *UNC5C*. A methylation score for at least one of the six candidate regions within these genes' promoters was present in over 95% of CRC cases, suggesting a viable detection method. In evaluating ccfDNA from 97 CRC patients and 62 control subjects, a difference in methylation and recovery signatures was observed. The combined score, integrating both methylation and recovery metrics, showed high diagnostic accuracy, evidenced by an area under the ROC curve of 0.90 (95% CI = 0.86 to 0.94). While correlating with tumor burden, this score gave early insight into disease progression in a small patient cohort. Our results suggest that DNA methylation in ccfDNA could serve as a sensitive biomarker for CRC, offering a less invasive and potentially more cost-effective approach to augment existing cancer detection and monitoring modalities, possibly supporting comprehensive genetic mutation profiling.

**Supplementary Information:**

The online version contains supplementary material available at 10.1186/s12943-023-01910-y.

## Introduction

Circulating cell-free DNA (ccfDNA) based liquid assays are emerging as promising noninvasive approaches for early cancer relapse detection and monitoring treatment effectiveness. Several observational studies involving patients with solid tumors have confirmed a very high risk of cancer recurrence when circulating tumor DNA (ctDNA) levels are detectable following a curative-intent therapy and those who did not receive further adjuvant treatment [1–3]. Recently, the DYNAMIC trial highlighted that the ctDNA-guided approach significantly reduced the use of adjuvant chemotherapy without increasing the risk of disease recurrence in patients with stage II colorectal cancer (CRC) [4].

While such liquid biopsy tests have shown promise in clinical settings for various solid malignancies, their analytical complexity and high expense make them challenging for a more comprehensive and routine adaptation in the clinic [5, 6]. Identifying unique somatic mutations through next-generation sequencing (NGS) analysis of a tumor tissue specimen is arduous, time-consuming, and challenging to standardize across different analytical platforms [7, 8]. Simultaneously, targeted NGS panels without a priori knowledge of the patient-specific mutational profile remain challenging and require significant standardization [4, 6, 9, 10]. In other words, despite technological advances, the use of ctDNA biomarkers for early detection of cancer recurrence still requires further research to improve the overall diagnostic accuracy while minimizing false-positive signals from clonal hematopoiesis, library preparation, and sequencing errors [7]. Unsurprisingly, ongoing research focused on enhancing the sensitivity and specificity of these liquid biopsy assays to make them more widely available in clinical settings [7].

Aberrant DNA methylation is a common and early event in cancer development [11]. While cancer patients may have unique methylation patterns, specific DNA methylation changes are found within each type of cancer. For instance, hypermethylation of *SEPT9* or *SFRP2* is a frequent event in CRC and may act as a universal biomarker for monitoring patients with CRC [12, 13]. DNA methylation biomarkers have demonstrated potential in areas such as tumor screening, prognosis assessment, evaluation of therapeutic efficacy, and personalized treatment, as previously demonstrated by us and other laboratories [13].

Building upon this emerging evidence, in this study, we examined whether detecting aberrant cancer-specific methylation patterns in ccfDNA, using multiple tumor-specific methylated loci, can serve as effective biomarkers for predicting tumor burden and monitoring the therapeutic response to antitumor therapies in CRC.

## Findings and discussion

### *Aberrant methylation in* EFEMP1, SFRP2,* and* UNC5C* promoters in CRC specimens*

**Fig. 1:**
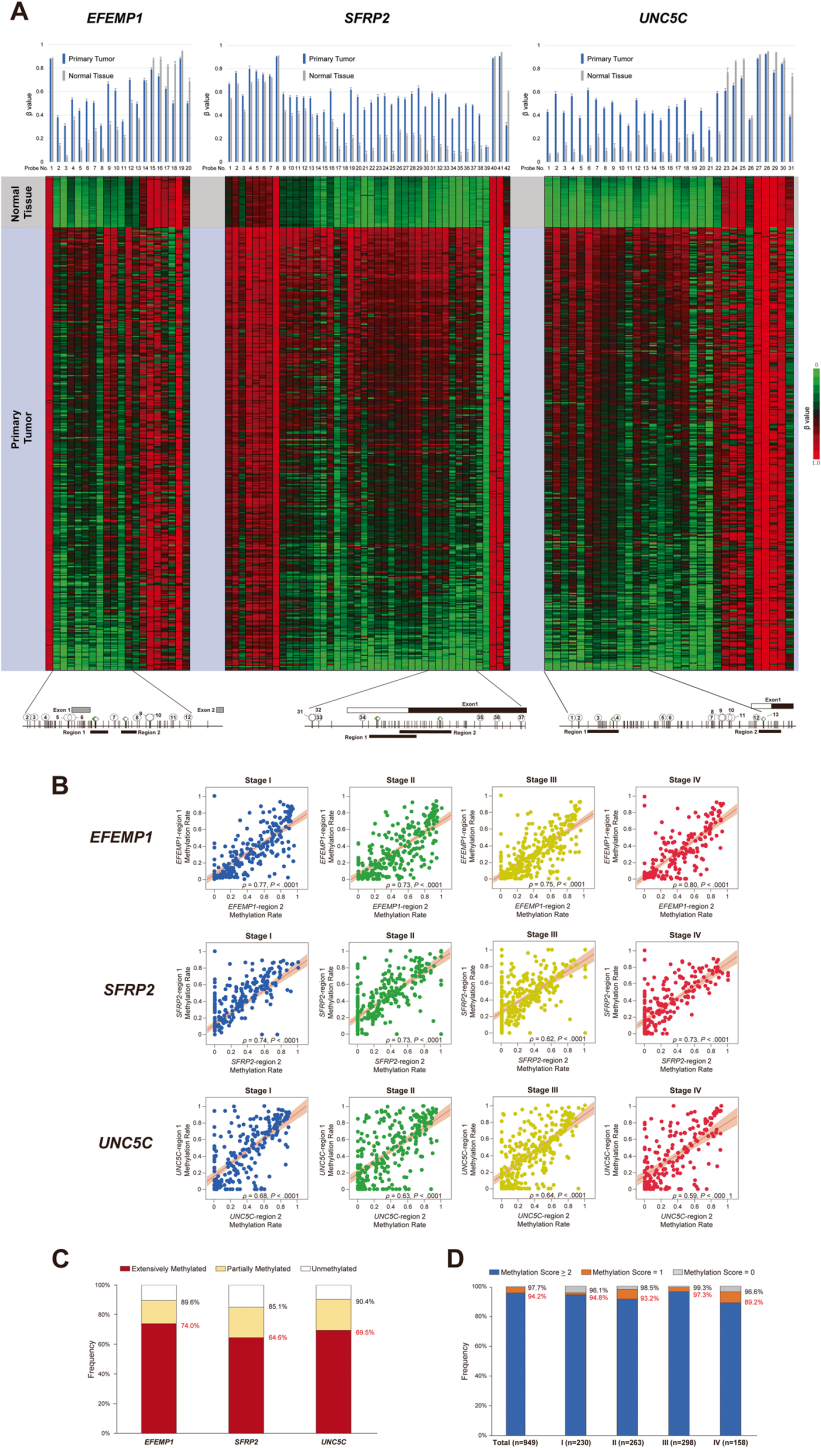
Summary of Methylation Profiles in *EFEMP1*, *SFRP2*, and *UNC5C* of TCGA-COADREAD Data and additional 949 CRC Samples. **A** Methylation Profiles in *EFEMP1*, *SFRP2*, and *UNC5C* of TCGA-COADREAD Data. The bar graphs above compare the average *β* values of probes in each gene between tumors and normal mucosa. The T-bars represent the standard deviations. The central heatmaps display the *β* values for each probe in 395 CRC samples and 45 normal mucosa samples. The lower schematics show the positions of CpG sites in the promoter regions of each gene, the probe locations, and the PCR products (black bars of Region 1 and Region 2) analyzed by COBRA and Hi-SA. Diamonds indicate the recognition sites of the HhaI restriction enzyme, and the circled numbers indicate the probe numbers for each gene. **B** Associations of methylation ratio between region 1 and region 2 of *EFEMP1*, *SFRP2*, and *UNC5C* in stages I, II, III, and IV. The red line represents the regression line, and the light red indicates the 95% confidence interval. The Wilcoxon rank-sum test was evaluated to estimate the* P* values. **C** Frequencies of extensive, partial methylation, and unmethylation in *EFEMP1*, *SFRP2*, and *UNC5C.* **D** Frequencies of methylation score 2 or more, 1 or more according to the UICC stage

Please refer to Supplementary Methods for the materials and methods of this study. Study participants were shown in Supplementary Fig. S1. Before selecting our biomarkers, we screened candidate markers that exhibited methylation positivity (5% or more methylation levels) in over 60% of CRC cases, analysing 16 genes or loci referenced in our previous studies. Through this process, *3OST2*, *EFEMP1*, *HPP1*, *RASSF2*, *SFRP2*, and *UNC5C* emerged as potential candidates [14–16]. However, our preliminary analysis revealed that mean methylation levels of 5.8% for *3OST2* and 5.6% for *HPP1* were present in the normal mucosa (analysis included 208 normal mucosa samples).*RASSF2*, while displaying a partial or extensive methylation frequency of nearly 80%, was excluded from the selected marker set due to its low extensive methylation frequency of 26%. Therefore, we chose *EFEMP1*, *SFRP2*, and *UNC5C* as the biomarkers for our methylation assay. To confirm whether the methylation rate in these biomarkers increased in cancer specimens, we initially examined their methylation profiles in 395 CRC and 45 normal colonic tissues using DNA methylation array data from TCGA databases (Fig. 1A and Supplementary Tables S1, S2, S3). The mean *β* value of probes located within the promoter region in the three genes was significantly higher in cancer specimens than in the normal colonic mucosa. Notably, our previous reports showed that the methylation spread within the promoter of tumor suppressor genes correlates with cancer progression [13], and the three candidate genes showed a predisposition for methylated CpG sites within their specific promoter regions in the TCGA dataset.

To assess the reproducibility of the aberrant DNA methylation features in *EFEMP1*, *SFRP2*, and *UNC5C* genes and their association with critical clinicopathological characteristics, we investigated the ratio and frequency of methylation in two distinct regions of each gene promoter in a cohort of 949 CRC specimens obtained from resected tissues using the Combined bisulfite restriction analysis (COBRA) with fluorescent dyes.

In the 949 CRC tissues analyzed, the mean methylation ratio was 0.32 (95% confidence interval [CI] = 0.30 to 0.33) for *EFEMP1*-region 1, 0.41 (95% CI = 0.39 to 0.43) for *EFEMP1*-region 2, 0.39 (95% CI = 0.38 to 0.41) for *SFRP2*-region 1, 0.28 (95% CI = 0.26 to 0.29) for *SFRP2*-region 2, 0.42 (95% CI = 0.40 to 0.44) for *UNC5C*-region 1, and 0.36 (95% CI = 0.34 to 0.37) for *UNC5C*-region 2. Strong-positive correlations were observed between methylation in regions 1 and 2 of each gene across UICC stages I to IV (Fig. 1B).

Next, we determined methylation positivity based on the ratio of methylated CpG sites at 0.05 or more, consistent with previous studies. The associations between clinical characteristics and methylation status in each locus are listed using this binary categorization in Supplementary Table S4. The 949 CRC cohort included microsatellite instability-high (both Lynch syndrome and sporadic tumors), tumors with *BRAF* V600E mutation, *POLE* mutation, or *KRAS* mutations. Aberrant methylation in the six loci was consistently observed, irrespective of such genetic mutational features.

Furthermore, to examine the critical methylation spreading pattern within the *EFEMP1*, *SFRP2*, and *UNC5C* genes, we evaluated the frequency of partial methylation (i.e., affecting either region 1 or 2) and extensive methylation (i.e., affecting both regions 1 and 2), which are presented in Fig. 1C and Supplementary Table S5.

We assigned each CRC sample a numerical score reflecting the number of methylated genes to enhance further the ability to predict CRC using the methylation status of *EFEMP1, SFRP2,* and *UNC5C*. The associations between clinical characteristics and the methylation score are listed in Supplementary Table S6.

The methylation score of one or more genes was observed in 97.7% and that of two or more regions in 94.2% of CRCs. Upon closer examination, a methylation score of one or more regions was observed in 96.1% of stage I, 98.5% in stage II, 99.3% in stage III, and 96.6% of stage IV cancers. Similarly, a methylation score of two or more methylated loci was observed in 94.8% of stage I, 93.2% in stage II, 97.3% in stage III, and 89.2% in stage IV cancers (Fig. 1D). In other words, methylation status of the six candidate regions (*EFEMP1*, *SFRP2*, and *UNC5C*, as well as the two regions within each gene) can be used to assign a numerical score to each CRC sample, which is associated with the number of methylated genes. This methylation score is a potential predictor of CRC and shows a high frequency of methylation across all stages of CRC.

### Methylation and recovery signatures in ccfDNA in subjects with or without cancer burden

**Fig. 2:**
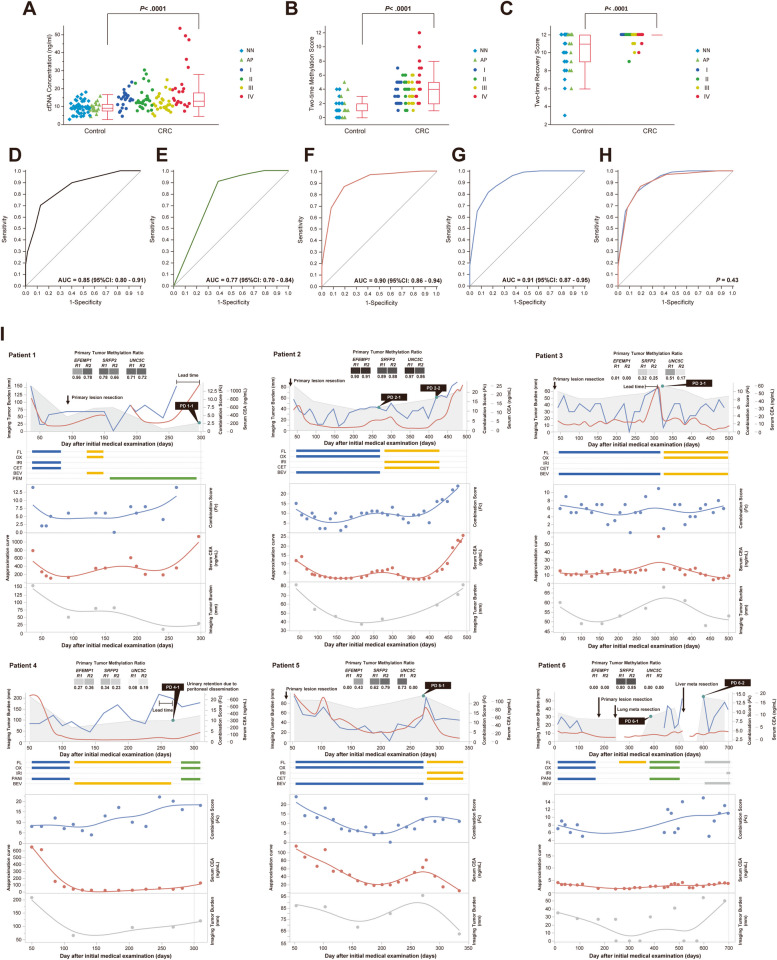
Summary of ccfDNA Methylatyion Analyzes. **A** The concentration of ccfDNA, **B** The two-time methylation score, and **C** The two-time recovery score were analyzed in both CRC patients and control subjects (Control). The box plot diagrams show the median as a horizontal line within each box, the interquartile ranges as the box limits, and the maximum and minimum values as the whiskers. *P* values were calculated using Dunn's test. ROC analyzes were performed to distinguish between CRC patients (*n* = 97) and control subjects (*n* = 62) using **D** The two-time methylation score, **E** The two-time recovery score, **F** The combination score (*Fc*), and **G** The fivefold cross-validation evaluated by both recovery and methylation scores. **H** No statistical difference was found between the ROC curve obtained using the combination score (*Fc*) and the fivefold cross-validation. **I** Examples of the combination Score (*Fc*), serum CEA Level, and the sum of the maximum equator of metastatic lesions (the imaging tumor burden) during the clinical course of six mCRC patients receiving systemic chemotherapy and surgical resections. The primary tumor methylation profile shows the methylation ratios at six loci, with R1 and R2 representing Region 1 and Region 2, respectively. Squares denote the heat map of methylation ratios, with black indicating a ratio of 1.0 and white indicating a ratio of 0.0. The number beneath each square represents the methylation ratio. The blue line in the top graph represents the combination score (*Fc*), while the red line represents the serum CEA level. The gray shade represents the imaging tumor burden. The middle horizontal bar denotes the treatment agent used, with blue, yellow, green, and gray bars representing the first, second, third, and fourth lines, respectively. FL: Fluoropyrimidine; OX: Oxaliplatin; IRI: Irinotecan; CET: Cetuximab; BEV: Bevacizumab; PEM: Pembrolizumab; PANI: Panitumumab. The graphs below show the approximation curves estimated by the combination score (*Fc*) (blue line), the serum CEA level (red line), and the imaging tumor burden (gray line), respectively

Supplementary Table S7 summarizes the clinical characteristics of the 97 patients with CRC and 62 control subjects for ccfDNA methylation analyzes. Before investigating methylation features, we evaluated the association between ccfDNA concentration in patients with CRC and control subjects. The mean concentration of ccfDNA was significantly higher in the CRC patients than in the control subjects (the mean concentrations in the CRC patients and the control subjects were 15.2 ng/ml [95% CI = 13.5–16.9] and 9.5 ng/ml [95% CI = 8.7–10.3], respectively, *P* < 0.0001, Fig. 2A).

Next, to evaluate the methylation status in ccfDNA, a multiplex PCR strategy known as the High-sensitive assay for bisulfite DNA (Hi-SA) was used to retrieve methylated alleles from cancer cells secreted or discharged [13]. Supplementary Fig. S2 shows a panel of representative Hi-SA results for ccfDNA. Using this approach, we investigated whether the methylation and recovery signatures in ccfDNA could differentiate between patients with CRC before surgical resection and control subjects without any radiographic evidence of cancer burden. Methylation positivity was defined as the rate of methylated CpG sites at 0.01 (1.0%) or greater on Hi-SA in ccfDNA.

A known limitation of liquid biopsy analysis is the proportion of ctDNA in ccfDNA [17]. This proportion is considered very low when the tumors are small and localized; however, this proportion increases as tumors grow and metastasize[17, 18]. In this study, the concentration of ccfDNA in patients with CRC was also significantly higher than that in the control subjects. Among CRC patients, Stage IV patients exhibited the highest ccfDNA concentration. However, this measurement could only predict the amount of ccfDNA, not that of ctDNA. Generally, NGS-based methods overcome the challenges posed by low ctDNA proportions by detecting tumor-specific mutations through increased depth of coverage.

To improve the detection capabilities of ctDNA features using DNA methylation-based liquid biopsy, we performed Hi-SA twice per blood sample to ensure reproducibility and enhance the detection capacity rather than relying solely on depth coverage in NGS. As anticipated, the first and second methylation ratios displayed a statistically significant positive correlation across all six loci, demonstrating reproducibility (Supplementary Fig. S3). Additionally, the recovery score—a marker reflecting the amount of DNA not fragmented by apoptosis [13]—was significantly higher in CRC patients compared to the control subjects (15.8 [95% CI = 15.5–16.1] vs. 14.1 [95% CI = 13.7–14.5] for recovery score, *P* < 0.0001; Figs. 2B and C).

We stratified 62 control subjects into two subgroups: 46 subjects with no evidence of neoplastic disease (NN) and 16 with colorectal adenomatous polyps (AP) as determined by colonoscopy. We also stratified the group of 97 CRC patients into different UICC stages, with 23, 26, 27, and 21 CRC patients diagnosed at stages I, II, III, and IV after surgical resection. Although the number of samples for analysis was small when performing a Dunn test based on the merger rank with NN as the control group, the mean concentration of ccfDNA did not show a significant difference between NN and AP or between NN and Stage III. However, a significant difference was observed between NN and Stages I, II, and IV (Supplementary Fig. S4A). In contrast, the mean two-time methylation and recovery scores of ccfDNA did not significantly differ between NN and AP. However, significant differences were observed between NN and all stages of CRC patients (Supplementary Figs. S4B and C).

Given the importance of the recovery score, we developed the combination score (*Fc*) by integrating it with the methylation score. The combination score (*Fc*) was statistically evaluated by the diagnostic performance of the two-time methylation score and the two-time recovery score: *Fc* = *β1* × (two-time methylation score) + *β2* × (two-time recovery score), where *β1* denotes the parameter estimate for the two-time methylation score obtained from logistic regression, and *β2* denotes the two-time recovery score. The parameter estimate for the two-time methylation score (*β1*) was 0.95 (95% CI = 0.60 to 1.36, *P* < 0.0001), and that of the two-time recovery score (*β2*) was 0.99 (95% CI = 0.53 to 1.60, *P* = 0.0002). Thus, we fixed both* β1* and *β2* as 1.0 to further examine ccfDNA analysis. The ROC curves illustrate the fraction of true-positive results (sensitivity) and false-positive results (1-specificity) for various cut-off levels of the two-time methylation score, the two-time recovery score, and the combination score (*Fc*) (Figs. 2D-F and Supplementary Table S8). The AUCs for the two-time methylation score, the two-time recovery score, and the combination score (*Fc*) were 0.85 (95% CI = 0.80 to 0.91), 0.77 (95% CI = 0.70 to 0.84), and 0.90 (95% CI = 0.86 to 0.94), respectively. Next, we conducted five-fold cross-validation using the methylation and recovery scores collected twice from the 159 cases to examine whether the combination score (*Fc*) possessed generalization capabilities. The ROC curve using five-fold cross-validation on the same cohort had an AUC of 0.91 (95% CI = 0.87 to 0.95, Fig. 2G), and we found no statistical difference between the combination score (*Fc*) and the five-fold cross-validation (Fig. 2H; *P* = 0.43).

As methylation in the six loci of three genes was commonly observed in all stages of CRC, regardless of clinicopathological or molecular features, we determined whether the combination score (*Fc*) could effectively monitor tumor response to systemic chemotherapies. We reviewed the combination score (*Fc*) in 126 blood samples obtained from six metastatic CRC (mCRC) patients undergoing systemic chemotherapies to test this. Additionally, methylation ratios in the six loci were confirmed in biopsy samples taken from patients' primary tumors before treatment. Figure 2I shows the clinical course of six patients with the combination score (*Fc*), serum CEA level, the sum of the maximum equator of metastatic lesions (the imaging tumor burden), the methylation ratio found in the primary tumor in six loci, and the changes in chemotherapy regimens. Detailed clinical information for the patients is listed in Supplementary Table S9.

All six mCRC patients experienced progressive disease (PD) either radiologically or clinically. The trend of the approximation curves by the combination score (*Fc*) in patients 1, 2, 3, and 5 were similar to those of the serum CEA levels. In contrast, there was a divergence between the trends of approximation curves generated by the combination score (*Fc*) and those of the serum CEA levels observed in patients 4 and 6. However, the trends of approximation curves estimated by the imaging tumor burden resembled those of the combination score (*Fc*) rather than those of the serum CEA levels in patients 4 and 6. Especially in patient 6, serum CEA levels were within normal limits throughout the chemotherapy period. The combination score (*Fc*) sometimes increased before radiological or clinical PD. For example, patients 1 and 3 had a lead time of the combination score (*Fc*) of 35 and 49 days before radiological PD, respectively. Patient 4 had an increase in the combination score (*Fc*) 21 days before clinical PD. Therefore, our data suggest the potential of the combination score (*Fc*) to indicate the therapeutic response early during treatment and offer a new surrogate biomarker for predicting tumor dynamics.

The limitations of this study include the following: the biomarker set selected in this study was chosen based on the observation of methylation in more than 95% of CRC tissues with at least one methylated site. The advantage of this biomarker set is that it may predict minimal residual tumors without prior analyzes, such as identifying cancer-specific mutations in the primary tumor. It may help to confirm the effectiveness of chemotherapy in patients who do not exhibit elevated tumor markers, such as CEA. However, this assay does not detect acquired mutations or intrinsic resistance. Furthermore, the analysis of CRC tissues indicates that not all cancer cells exhibit methylation according to the methylation rate, leaving intra-tumor heterogeneity unaddressed. Since this biomarker set is also widely methylated in various cancer types besides CRC, caution is necessary for primary cancer screening [19, 20]. Determining whether this assay can predict postoperative recurrence in patients undergoing curative resection for CRC remains a future challenge. Moreover, our study lacks specific DNA methylation data from lifelong cancer-free control blood samples, particularly in older adults. Nevertheless, compared to multi-targeted genetic approaches, this assay may offer a more accessible and cost-effective method for monitoring minimal residual disease and treatment response.

In conclusion, this study underscores the potential of cancer-specific DNA methylation in ccfDNA as a sensitive biomarker for monitoring therapeutic response and for liquid biopsy detection in CRC patients. Although COBRA/Hi-SA offers a cost-effective alternative for methylation detection, we recognize the potential of digital PCR (dPCR) for its analytical sensitivity and absolute quantification. The benefits and challenges of implementing dPCR in clinical settings, especially for differentiating between densely and sporadically methylated tissue, will be a crucial aspect of our continued and future research. Despite some limitations, this approach could provide a more accessible and cost-effective means to monitor minimal residual disease and treatment response, possibly supplementing comprehensive genetic mutation profiling. Further investigation is required to fully understand its applicability in diverse clinical contexts.

### Supplementary Information

**Additional file 1:** **Fig. S1.** Study Flow Charts. **Fig. S2.** Representative Hi-SA results for ccfDNA. **Fig. S3.** Association between the first and second methylation rates in two independent Hi-SA analyses. **Fig. S4.** Summary of Blood Sample Analyses. **Table S1.** The comparison of the mean beta value between tumor and normal mucosa on *EFEMP1* locus. **Table S2.** The comparison of the mean beta value between tumor and normal mucosa on *SFRP2* locus. **Table S3.** The comparison of the mean beta value between tumor and normal mucosa on *UNC5C* locus. **Table S4.** The associations between clinical characteristics and methylation status in each locus. **Table S5.** Clinical feature of spreading methylation in *EFEMP1*, *SFRP2*, and *UNC5C*. **Table S6.** Associations between clinical characteristics and the methylation score. **Table S7.** Clinical characteristics of CRC patients and control subjects for ccfDNA methylation analyses. **Table S8.** The cut-off values of scores for sensitivity in patients with CRC who are correctly identified as having the disease (true positives), as well as their 1-specificity for the proportion of individuals without the disease who are incorrectly identified as having the disease (false positives). **Table S9.** Clinical information and methylation status in the three genes of the six mCRC patients. **Table S10.** Primer sets of the Combined bisulfite restriction analysis (COBRA) with fluorescence dyes and High-sensitive assay for bisulfite DNA (Hi-SA).

## Data Availability

All data generated or analyzed during this study were included in this article. The datasets used and analyzed in the current study are available from the corresponding author upon reasonable request.

## Abbreviations

AUC: Area under the curve
ccfDNA: Circulating cell-free DNA
CEA: Carcinoembryonic antigen
COBRA: Combined bisulfite restriction analysis
CRC: Colorectal cancer
CRT: Chemoradiotherapy
CT: Computed tomography
ctDNA: Circulating tumor DNA
Hi-SA: High-sensitive assay for bisulfite DNA
ESD: Endoscopic submucosal dissection
mCRC: Metastatic colorectal cancer
NGS: Next-generation sequencing
PD: Progressive disease
RECIST: Response Evaluation Criteria in Solid Tumors
ROC curve: Receiver operating characteristic curve
RT: Radiotherapy
TCGA: The Cancer Genome Atlas Project
UICC: Union for International Cancer Control
