# Supplementary material for: Circulating cell-free DNA methylation patterns as non-invasive biomarkers to monitor colorectal cancer treatment efficacy without referencing primary site mutation profiles
Source: Mol Cancer. 2024 Jan 3;23:1. doi: 10.1186/s12943-023-01910-y (PMC10762960; doi:10.1186/s12943-023-01910-y)
Supplement: Supplementary file 1 — Additional file 1: Fig. S1. Study Flow Charts. Fig. S2. Representative Hi-SA results for ccfDNA. Fig. S3. Association between the first and second methylation rates in two independent Hi-SA analyses. Fig. S4. Summary of Blood Sample Analyses. Table S1. The comparison of the mean beta value between tumor and normal mucosa on EFEMP1 locus. Table S2. The comparison of the mean beta value between tumor and normal mucosa on SFRP2 locus. Table S3. The comparison of the mean beta value between tumor and normal mucosa on UNC5C locus. Table S4. The associations between clinical characteristics and methylation status in each locus. Table S5. Clinical feature of spreading methylation in EFEMP1, SFRP2, and UNC5C. Table S6. Associations between clinical characteristics and the methylation score. Table S7. Clinical characteristics of CRC patients and control subjects for ccfDNA methylation analyses. Table S8. The cut-off values of scores for sensitivity in patients with CRC who are correctly identified as having the disease (true positives), as well as their 1-specificity for the proportion of individuals without the disease who are incorrectly identified as having the disease (false positives). Table S9. Clinical information and methylation status in the three genes of the six mCRC patients. Table S10. Primer sets of the Combined bisulfite restriction analysis (COBRA) with fluorescence dyes and High-sensitive assay for bisulfite DNA (Hi-SA). [file 12943_2023_1910_MOESM1_ESM.pdf]

Circulating cell-Free DNA methylation patterns as non-invasive biomarkers to monitor colorectal cancer treatment efficacy without referencing primary site mutation profiles

Supplementary Materials

Kazuya Yasui[1]*, Toshiaki Toshima[1, 2]*, Ryo Inada[2], Yuzo Umeda[1], Shuya Yano[3], Hiroaki Tanioka[3], Akihiro Nyuya[3], Toshiyoshi Fujiwara[1], Takeshi Yamada[4], Yoshio Naomoto[5], Ajay Goel[6#], and Takeshi Nagasaka[3#]

[1] Department of Gastroenterological Surgery, Okayama University Graduate School of Medicine, Dentistry and Pharmaceutical Sciences, Okayama 700-8558, Japan.

[2] Department of Gastroenterological Surgery, Kochi Health Sciences Centre, Kochi, 781-0111, Japan.

[3] Department of Clinical Oncology, Kawasaki Medical School, Kurashiki, 701-0912, Japan

[4] Department of Gastrointestinal and Hepato-Biliary-Pancreatic Surgery, Nippon Medical School, Tokyo, 113-8602, Japan.

[5] Department of General Surgery, Kawasaki Medical School, Okayama, 700-8505 Japan.

[6]Department of Molecular Diagnostics and Experimental Therapeutics, Beckman Research Institute of City of Hope, Biomedical Research Center, Monrovia, CA, 91016, USA

MATERIALS AND METHODS

Methylation analyses in publicly available datasets

We obtained DNA methylation profiles for The Cancer Genome Atlas Project (TCGA)-Colon and Rectal Cancer (COADREAD), which included data from 398 colorectal cancer (CRC) patient tissues and 45 normal colonic tissues, from the UCSC Xena database (http://xena.ucsc.edu/). The Infinium Human Methylation 450 Bead Chip microarrays (Illumina Inc.) were used for genome-wide methylation analysis. We excluded three patients with CRC from the analysis because these patients either had metastatic disease or had recurrent lesions, resulting in a final analysis of 395 CRCs and 45 normal colon tissues (**Fig. S1A**).

Patient specimens, DNA extraction, and mutational and methylation analyses in CRC tissues

A cohort of 1,104 CRC tissue specimens was collected from surgical resections performed between 1998 and 2017 at Okayama University Hospital in Japan. Of these, 99 CRC patients who received any therapeutic intervention before surgical resection were excluded from further analysis. Among the eligible 1,005 CRC patients, 56 cases had insufficient data for analyzing methylation status. Consequently, 949 CRC patients, corresponding to the remaining tissue specimens, were enrolled in this study and included for further analyses (**Fig. S1B**). DNA extraction from CRC tissues, followed by the bisulfite conversion, was performed, as described previously [1]. The pathogenic hotspot mutations within the proofreading domain of the *POLE* gene (exons 9, 13, and 14), mutations in the *KRAS* exon 2, and *BRAF* exon 15 (including codon 600), as well as the MSI status, were determined as reported previously [1, 2].

To quantify the population of methylated alleles within the *EFEMP1 (Fibulin-3)*, *SFRP2*, and *UNC5C* promoters in CRC tissue samples, we performed a modified combined bisulfite restriction analysis (COBRA) with fluorescence dyes to quantify the methylation density [2-4].

The primer sequences and restriction enzymes used for these analyses are listed in **Table S10**. The PCR products were digested with HhaI (New England BioLabs, Ipswitch, MA, USA) and loaded simultaneously onto a SeqStudio Genetic Analyzer (Thermo Fisher Scientific). The unique fluorescent PCR signal distinguished individual PCR products for each target, and their fragment length and the data were analyzed using GeneMapper software 5 (Applied Biosystems, Foster City, CA, USA). The ratio of methylated CpG sites (digested by restriction enzymes) was calculated by the balance between the restriction enzyme-cleaved PCR products and the total amount of PCR product in each locus.

Methylation positivity was defined as the ratio of methylated alleles at 0.05 (5.0%) or more. The number of markers methylated subsequently determined the methylation score. By this definition, the methylation score in the CRC sample ranged between 0 to 6.

Blood specimen collection from a prospective cohort of patients with CRC

We prospectively recruited CRC patients between 2020 and 2022 at the Kochi Medical Centre in Kochi, Japan (**Fig. S1C**). A cohort of 97 blood samples from patients with CRC patients was collected before surgical resection. For the control group, blood samples were obtained from 70 patients who underwent curative resection for Union for International Cancer Control (UICC) stage I to III CRC with no evidence of recurrence at least one year after surgical resection, as confirmed by computed tomography (CT) and with no corresponding elevation of serum carcinoembryonic antigen (CEA) levels (ng/mL) throughout the post-surgery duration between 2016 and 2018 at Okayama University Hospital. All control subjects underwent colonoscopy after blood collection. Of the 70 cases, eight were excluded from further analysis for the following reasons: two cases with anal squamous cell cancer treated after CRT, one case with residual tumor treated after Endoscopic Submucosal Dissection, one case without colonoscopy examination, one

case with metastasis detected by CT, and three cases with insufficient blood collection due to hemolysis. Finally, the remaining 62 cases were categorized as the control group. Of these 62 cases, 16 were confirmed to have adenomatous polyps in the colon and rectum by colonoscopy. Therefore, the 62 control subjects were divided into 46 patients with no neoplasia (NN) and 16 with adenomatous polyps (AP) (**Fig. S1D**).

To examine whether our methylation biomarkers could effectively monitor tumor response during chemotherapy treatment, 126 blood samples were collected from six metastatic CRC (mCRC) patients before each treatment cycle of systemic chemotherapy at Okayama University Hospital.

A cohort of 97 blood samples from patients with CRC and 62 control subjects were collected with PAXgene Blood ccfDNA tubes (Qiagen NV, Hilden, Netherlands). The blood specimens from six mCRC patients were collected with an EDTA collection tube before each chemotherapy treatment and processed for plasma isolation immediately after collection. According to the manufacturer's protocol, the plasma was separated from whole blood and centrifuged at 3,000 *g* for 15 minutes at 4°C. Prepared plasma samples were stored at −80 °C until use.

Evaluation of tumor burden by radiographical examination and blood CEA levels

The control subjects were evaluated for tumor recurrence events by CT at several time points, typically every 3 to 6 months after curative resection, as decided by the physician or prescribed by a clinical trial conducted at the hospital.

Tumor response to systemic chemotherapies for the six mCRC patients was evaluated based on CT or MRI scans every eight weeks, according to the Response Evaluation Criteria in Solid Tumors (RECIST) version 1.1. This study defined the imaging tumor burden as the sum of

the maximum equator of up to six metastatic lesions. Serum CEA levels were evaluated using blood collected at the same time as the blood used in this study by a diagnostic laboratory at Okayama University Hospital or Kochi Medical Centre.

Extraction and bisulfite conversion of cfDNA

The cfDNA in serum was extracted using a QIAamp MinElute ccfDNA Kit (Qiagen), and the concentration of cfDNA was evaluated using a Qubit 4 Fluorometer (Thermo Fisher Scientific, MA) with the dsDNA H.S. Assay Kit (high sensitivity, 0.2 to 100 ng) following the manufacturer's instructions. Bisulfite modification of cfDNA (total amount of cfDNA was 5 ng per sample) was performed using an EZ DNA Methylation-Lightning Kit (Zymo Research).

Detection of methylated and unmethylated alleles in circulating cell-free DNA

To assess the methylation status in ccfDNA, we adopted a high-sensitivity assay for bisulfite DNA (Hi-SA) to recover and confirm methylated alleles from cancer cells [4]. We optimized a multiplex-PCR strategy to recover all six loci in the *EFEMP1*, *SFRP2*, and *UNC5C* promoter simultaneously. Hi-SA can detect methylation ratios as low as 0.01 of methylated alleles per unmethylated allele using internal methylation-specific primers; hence, the methylation positivity was defined as the ratio of methylated CpG sites at 0.01 (1.0%) or more in ccfDNA. Similar to the fluorescent COBRA analysis of CRC tissues, a methylation score was given by the number of markers methylated in each case. Additionally, a recovery score was provided by the number of loci amplified from ccfDNA. When the sum of the fluorescence intensity of the methylated and unmethylated alleles exceeded 100, we judged that the target locus was successfully '*recovered*.' Thus, methylation and recovery scores ranged from 0 to 6 at a given time. Hi-SA was performed twice per blood sample to confirm the reproducibility and improve the detection

capacity. Thus, methylation and recovery scores ranged from 0 to 6 at one inspection and 0 to 12 at the sum of two-time analysis (two-time sum of methylation or recovery score).

Statistical Analysis

All statistical analyses were performed using JMP Genomics software (version 10.2; SAS Institute, Inc., Cary, NC, USA). The chi-squared test was used to examine the associations between categorical variables. The comparison of the mean beta (β) value between tumor and normal mucosa calculated from the TCGA database in each probe was analyzed by analysis of variance. The methylation ratios of the *EFEMP1*, *SFRP2*, and *UNC5C* promoters were analyzed as continuous and categorical variables. In analyzing CRC tissues, we assessed the relationship between methylation ratios in Region 1 and Region 2 of three genes by employing linear regression models. We calculated Spearman's rank correlation coefficients (ρ) to quantify this association. Furthermore, we assigned a numerical score to each CRC sample to represent the count of methylated loci. We used the Wilcoxon rank-sum test to compare the average methylation scores across different subgroups.

In the ccfDNA analysis, we employed linear regression models to estimate the relationship of methylation ratios at each locus between the first and second Hi-SA inspections. In addition, we determined the association of methylation rates at each locus between the two inspections by calculating Spearman's rank correlation coefficients (ρ). We also assigned a numerical score to each ccfDNA sample, representing the number of recovered and methylated loci. To perform nonparametric comparisons, we used the Dunn method for joint ranking, comparing ccfDNA samples with 62 control subjects or 46 NN subjects as the control group. This method calculates ranks for the entire dataset, not just the comparison pairs, resulting in *P* values reflecting a Bonferroni adjustment that Dunn's test reported.

We estimated the combination score (*Fc*), a function based on parameter estimates obtained from multiple logistic regression. The receiver operating characteristic (ROC) curve was plotted for potential cut-off values based on the two-time methylation score, the two-time recovery score, and the combination score (*Fc*) for the sensitivity of ccfDNA in patients with CRC. Similarly, the specificity was determined for control subjects. The area under the ROC curve (AUC) was measured to compare the screening efficiency of each score. The ROC curve for the combination score (*Fc*) was compared to that for the 5-fold cross-validation evaluated by both recovery and methylation scores, which gave identical results. A nonparametric approach was used to compare the AUCs, estimated by the combination score (Fc) and the 5-fold cross-validation [5]. All *P* values reported were calculated in two-sided tests, and values less than 0.05 were considered statistically significant.

References

1. Nagasaka T, Koi M, Kloor M, Gebert J, Vilkin A, Nishida N, Shin SK, Sasamoto H, Tanaka N, Matsubara N, et al: **Mutations in both KRAS and BRAF may contribute to the methylator phenotype in colon cancer.** *Gastroenterology* 2008, **134:**1950-1960, 1960 e1951.
2. Kawai T, Nyuya A, Mori Y, Tanaka T, Tanioka H, Yasui K, Toshima T, Taniguchi F, Shigeyasu K, Umeda Y, et al: **Clinical and epigenetic features of colorectal cancer patients with somatic POLE proofreading mutations.** *Clin Epigenetics* 2021, **13:**117.
3. Yoshida K, Nagasaka T, Umeda Y, Tanaka T, Kimura K, Taniguchi F, Fuji T, Shigeyasu K, Mori Y, Yanai H, et al: **Expansion of epigenetic alterations in EFEMP1 promoter predicts malignant formation in pancreatobiliary intraductal papillary mucinous neoplasms.** *J Cancer Res Clin Oncol* 2016, **142:**1557-1569.
4. Nagasaka T, Tanaka N, Cullings HM, Sun DS, Sasamoto H, Uchida T, Koi M, Nishida N, Naomoto Y, Boland CR, et al: **Analysis of fecal DNA methylation to detect gastrointestinal neoplasia.** *J Natl Cancer Inst* 2009, **101:**1244-1258.
5. DeLong ER, DeLong DM, Clarke-Pearson DL: **Comparing the areas under two or more correlated receiver operating characteristic curves: a nonparametric approach.** *Biometrics* 1988, **44:**837-845.

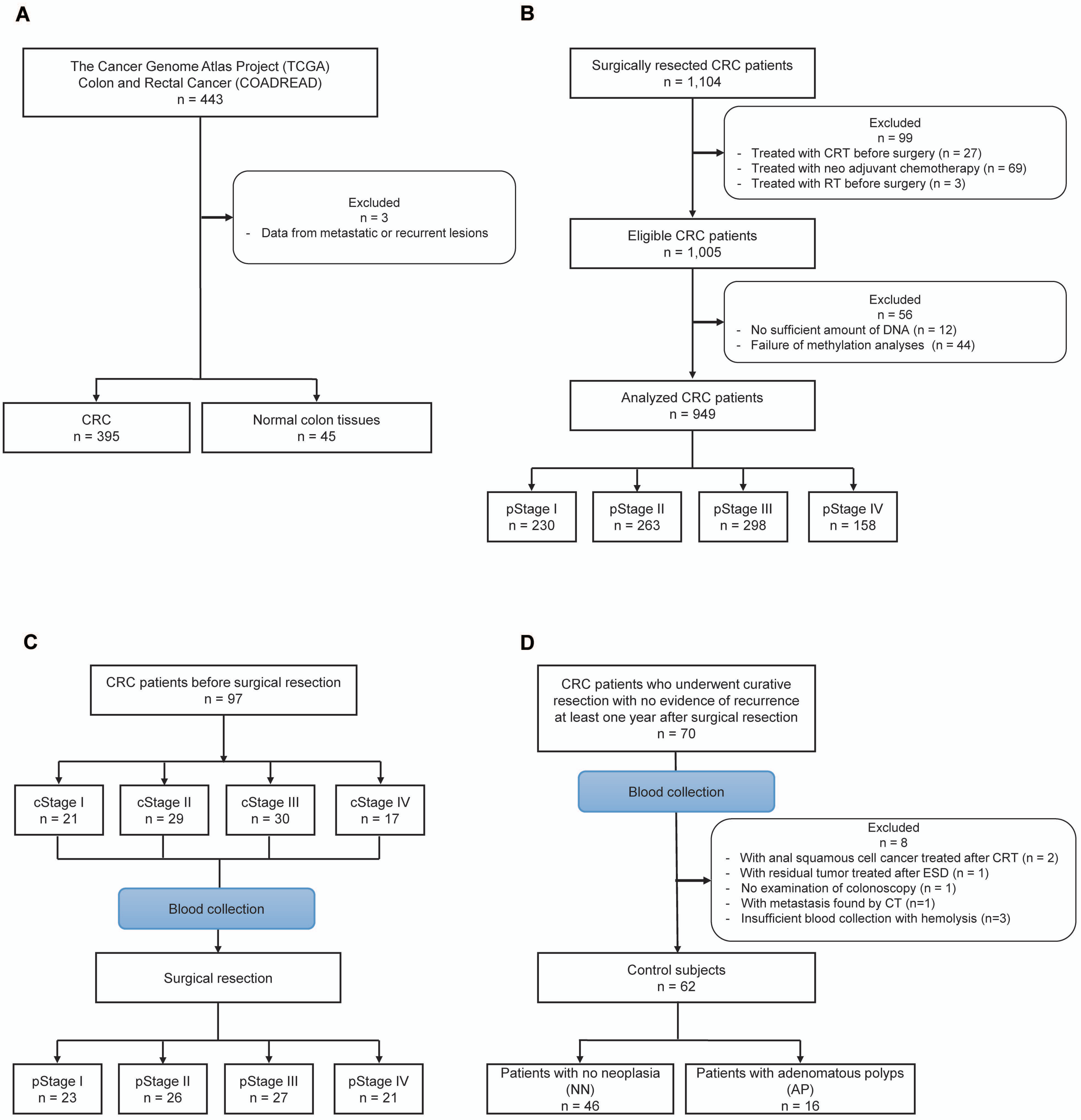
A
The Cancer Genome Atlas Project (TCGA)
Colon and Rectal Cancer (COADREAD)
n = 443
Excluded
n = 3
- Data from metastatic or recurrent lesions
CRC
n = 395
Normal colon tissues
n = 45
B
Surgically resected CRC patients
n = 1,104
Excluded
n = 99
- Treated with CRT before surgery (n = 27)
- Treated with neo adjuvant chemotherapy (n = 69)
- Treated with RT before surgery (n = 3)
Eligible CRC patients
n = 1,005
Excluded
n = 56
- No sufficient amount of DNA (n = 12)
- Failure of methylation analyses (n = 44)
Analyzed CRC patients
n = 949
pStage I
n = 230
pStage II
n = 263
pStage III
n = 298
pStage IV
n = 158
C
CRC patients before surgical resection
n = 97
cStage I
n = 21
cStage II
n = 29
cStage III
n = 30
cStage IV
n = 17
Blood collection
Surgical resection
pStage I
n = 23
pStage II
n = 26
pStage III
n = 27
pStage IV
n = 21
D
CRC patients who underwent curative
resection with no evidence of recurrence
at least one year after surgical resection
n = 70
Blood collection
Excluded
n = 8
- With anal squamous cell cancer treated after CRT (n = 2)
- With residual tumor treated after ESD (n = 1)
- No examination of colonoscopy (n = 1)
- With metastasis found by CT (n=1)
- Insufficient blood collection with hemolysis (n=3)
Control subjects
n = 62
Patients with no neoplasia
(NN)
n = 46
Patients with adenomatous polyps
(AP)
n = 16

Fig. S1: Study Flow Charts.

(A) Samples of TCGA-COADREAD were analyzed, which included 398 CRCs and 45 normal colon tissues, from UCSC Xena (http://xena.ucsc.edu/). (B) CRC tissue sample. The pathological stage (pStage) is a classification based on pathological findings. CRT denotes chemoradiotherapy. RT denotes radiotherapy. (C) Blood samples of patients with CRC patients. The clinical stage (cStage) is a classification based on pre-treatment clinical findings. (D) Blood samples of subjects without malignant tumor burden. ESD denotes endoscopic submucosal dissection. CT denotes computed tomography.

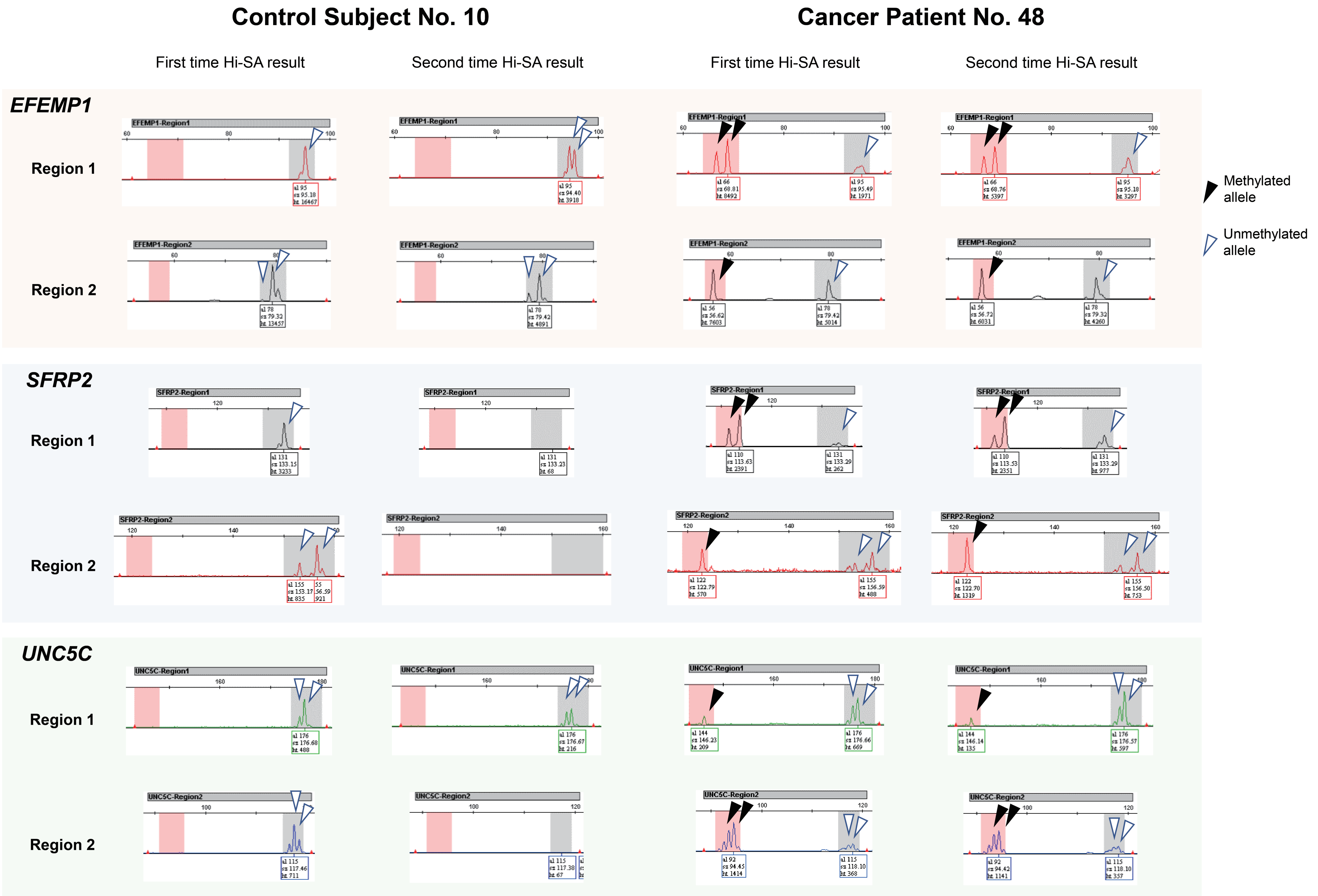
Control Subject No. 10
Cancer Patient No. 48
First time Hi-SA result
Second time Hi-SA result
First time Hi-SA result
Second time Hi-SA result
EFEMP1
Region 1
Region 2
SFRP2
Region 1
Region 2
UNC5C
Region 1
Region 2
Methylated allele
Unmethylated allele

Fig. S2. **Representative Hi-SA results for ccfDNA.**

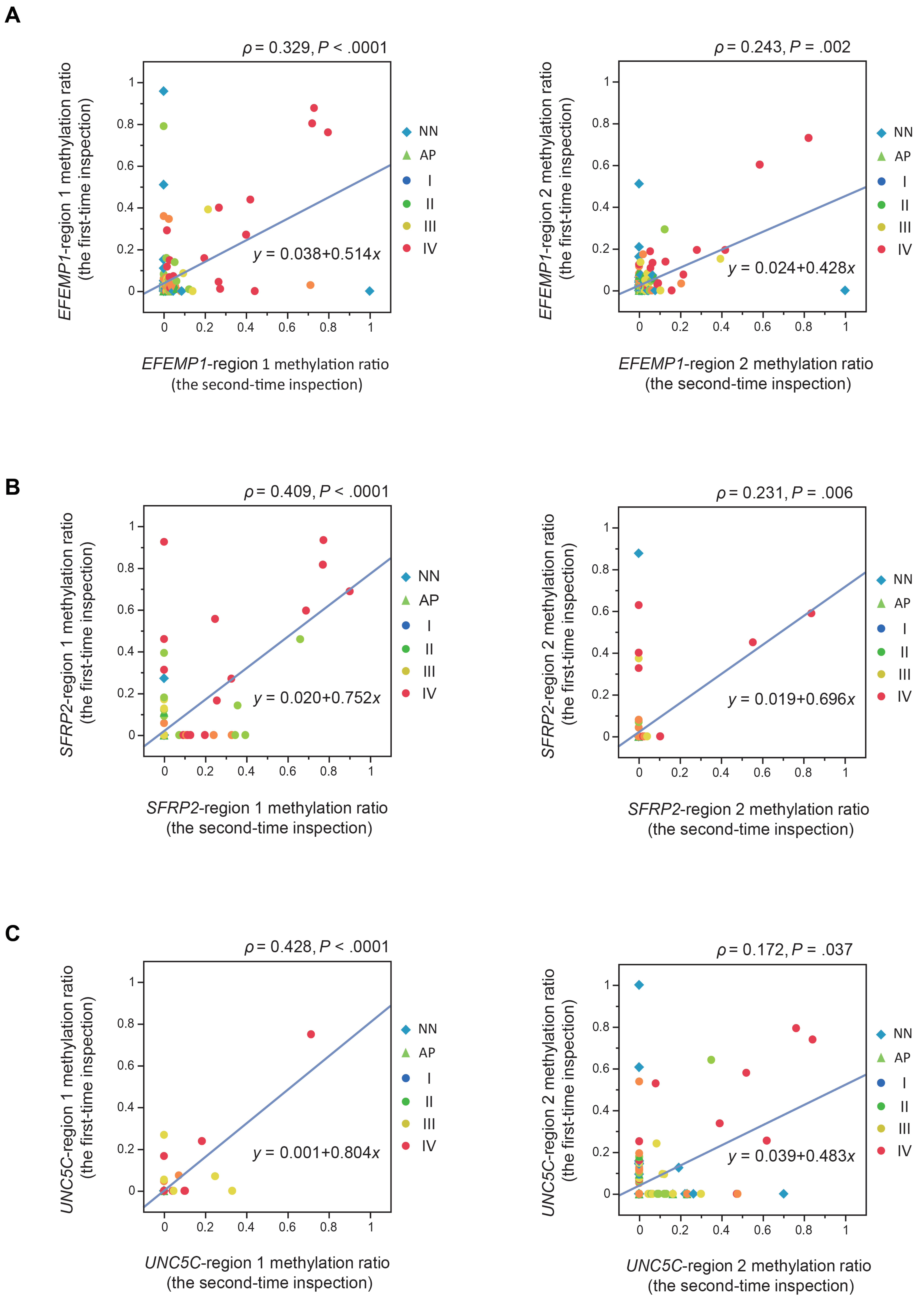
A
ρ = 0.329, P < .0001
y = 0.038+0.514x
EFEMP1-region 1 methylation ratio (the first-time inspection)
EFEMP1-region 1 methylation ratio (the second-time inspection)
ρ = 0.243, P = .002
y = 0.024+0.428x
EFEMP1-region 2 methylation ratio (the first-time inspection)
EFEMP1-region 2 methylation ratio (the second-time inspection)
B
ρ = 0.409, P < .0001
y = 0.020+0.752x
SFRP2-region 1 methylation ratio (the first-time inspection)
SFRP2-region 1 methylation ratio (the second-time inspection)
ρ = 0.231, P = .006
y = 0.019+0.696x
SFRP2-region 2 methylation ratio (the first-time inspection)
SFRP2-region 2 methylation ratio (the second-time inspection)
C
ρ = 0.428, P < .0001
y = 0.001+0.804x
UNC5C-region 1 methylation ratio (the first-time inspection)
UNC5C-region 1 methylation ratio (the second-time inspection)
ρ = 0.172, P = .037
y = 0.039+0.483x
UNC5C-region 2 methylation ratio (the first-time inspection)
UNC5C-region 2 methylation ratio (the second-time inspection)
NN
AP
I
II
III
IV

Fig. S3. Association between the first and second methylation rates in two independent Hi-SA analyses.

Linear regression models were employed to estimate the relationship between methylation ratios at each locus in the first and second Hi-SA inspections. The association between methylation rates at each locus in the two inspections was determined by calculating Spearman's rank correlation coefficients (ρ). In the two independent Hi-SA analyses, the first and second methylation rates demonstrated a statistically significant positive correlation across all six loci.

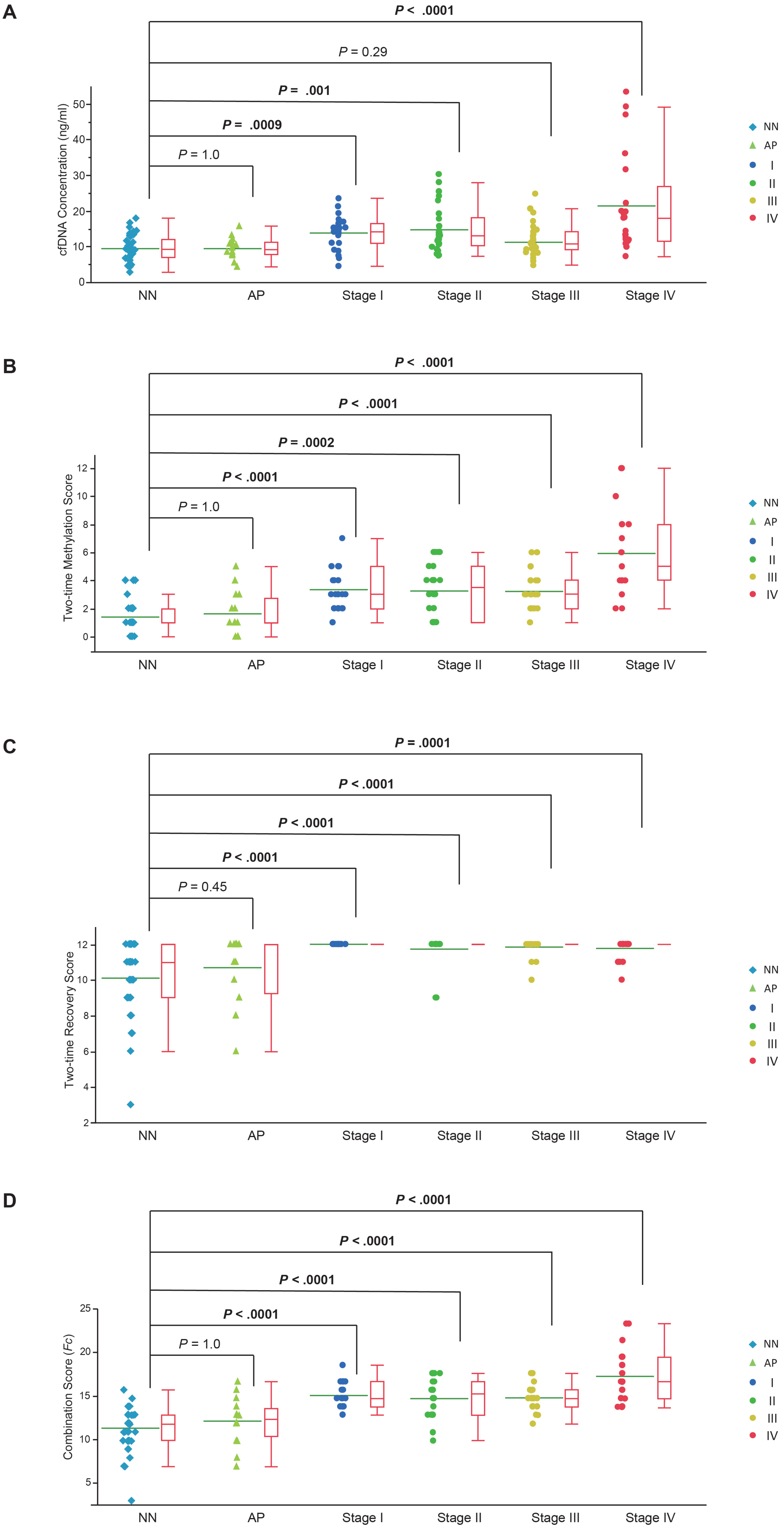

A
P < .0001
P = 0.29
P = .001
P = .0009
P = 1.0
cfDNA Concentration (ng/ml)
NN
AP
Stage I
Stage II
Stage III
Stage IV
NN
AP
I
II
III
IV
B
P < .0001
P < .0001
P = .0002
P < .0001
P = 1.0
Two-time Methylation Score
C
P = .0001
P < .0001
P < .0001
P < .0001
P = 0.45
Two-time Recovery Score
D
P < .0001
P < .0001
P < .0001
P < .0001
P = 1.0
Combination Score (Fc)

Fig. S4. Summary of Blood Sample Analyses.

(A) The concentration of ccfDNA, **(B)** the two-time methylation score, **(C)** the two-time recovery score, and **(D)** the combination score (*Fc*) was analyzed between CRC patients divided into the stages, subjects with colorectal adenomatous polyps (AP) and subjects with no evidence of neoplastic disease (NN). The box plot diagrams show the median as a horizontal line within each box, the interquartile ranges as the box limits, and the maximum and minimum values as the whiskers. *P* values were calculated using Dunn's test.

Table S1. The comparison of the mean beta value between tumor and normal mucosa on *EFEMP1* locus.

			EFEMP1						
Probe name	Array ID	Genomic Coordinate	Tumor mean beta value (N = 395)			Normal beta value (N = 45)			*P* value
			Mean	Lower 95%CI	Upper 95%CI	Mean	Lower 95%CI	Upper 95%CI	
probe1	cg25412594	56152003	0.878589	0.874973	0.882205	0.8782	0.8697	0.8867	0.945
probe2	cg25968367	56151179	0.380703	0.364875	0.396532	0.141307	0.124599	0.158015	< .0001
	cg19702569	56151165		N/A			N/A		
probe3	cg03817671	56151139	0.308045	0.288352	0.327739	0.044144	0.03471	0.05358	< .0001
probe4	cg16118212	56151071	0.532859	0.517923	0.547796	0.356702	0.331652	0.38175	< .0001
probe5	cg05168033	56150942	0.435482	0.417098	0.453866	0.098916	0.079246	0.118585	< .0001
probe6	cg08130988	56150925	0.51684	0.497935	0.535745	0.162184	0.140063	0.184306	< .0001
probe7	cg24719005	56150675	0.504025	0.489599	0.518451	0.26144	0.23976	0.28312	< .0001
probe8	cg25046074	56150549	0.308437	0.292624	0.32425	0.1029	0.092405	0.113395	< .0001
probe9	cg05385513	56150478	0.665669	0.647025	0.684314	0.317527	0.291309	0.343744	< .0001
probe10	cg16100120	56150475	0.607292	0.588651	0.625933	0.270722	0.247003	0.294441	< .0001
probe11	cg03122624	56150341	0.343631	0.328476	0.358787	0.212411	0.197593	0.22723	< .0001
probe12	cg20786074	56150255	0.699775	0.685795	0.7137545	0.501751	0.478074	0.525429	< .0001
probe13	cg25711779	56149825	0.496072	0.482014	0.510131	0.362338	0.352424	0.372252	< .0001
probe14	cg23260993	56149464	0.693801	0.674857	0.712745	0.678953	0.661761	0.696146	0.6054
probe15	cg00574639	56146874	0.788285	0.772089	0.8044811	0.871027	0.858327	0.883726	0.0008
probe16	cg02626897	56127942	0.730002	0.710804	0.7492322	0.878487	0.864	0.892974	< .0001
probe17	cg25268863	56116062	0.623299	0.605705	0.6408943	0.815969	0.802589	0.829349	< .0001
probe18	cg05140065	56113478	0.498686	0.476769	0.5206024	0.833111	0.812728	0.854949	< .0001
probe19	cg20052010	56108750	0.879991	0.867783	0.8921986	0.939396	0.931967	0.946824	0.0014
probe20	cg16947612	56103666	0.500012	0.484074	0.5159505	0.686191	0.657719	0.714663	< .0001

N/A denotes not available.

All *P* values was analyzed by analysis of variance.

Table S2. The comparison of the mean beta value between tumor and normal mucosa on *SFRP2* locus.

			SFRP2						
Probe name	Array ID	Genomic Coordinate	Tumor mean beta value			Normal beta value			*P* value
			Mean	Lower 95%CI	Upper 95%CI	Mean	Lower 95%CI	Upper 95%CI	
	cg04959480	154711675		N/A			N/A		
probe1	cg10663078	154711629	0.668056	0.657398	0.678715	0.535618	0.523356	0.547879	< .0001
probe2	cg24968721	154711620	0.764433	0.754894	0.773972	0.658793	0.645092	0.672495	< .0001
probe3	cg10790791	154711597	0.565885	0.550969	0.580801	0.428789	0.415816	0.441761	< .0001
probe4	cg05050042	154711563	0.797325	0.784383	0.810268	0.669744	0.644363	0.695126	< .0001
probe5	cg05241277	154711519	0.775345	0.764302	0.786387	0.6972	0.684379	0.710021	< .0001
probe6	cg13732865	154711512	0.74913	0.73808	0.760182	0.673927	0.660236	0.687628	< .0001
probe7	cg21657059	154711470	0.740347	0.732458	0.748237	0.716171	0.706103	0.72624	0.0449
probe8	cg01298731	154711457	0.901209	0.896563	0.905855	0.904562	0.897721	0.911404	0.6369
	cg01821854	154711347		N/A			N/A		
	cg21630608	154711304		N/A			N/A		
probe9	cg20727217	154711280	0.580563	0.567396	0.593731	0.426362	0.410798	0.441927	< .0001
probe10	cg00705808	154711183	0.554923	0.536507	0.573339	0.39678	0.381573	0.411987	< .0001
probe11	cg13357229	154711171	0.556456	0.538091	0.574822	0.413193	0.398835	0.427552	< .0001
probe12	cg23502475	154711115	0.550134	0.536022	0.564247	0.44052	0.43018	0.45086	< .0001
probe13	cg24241928	154711099	0.547205	0.529833	0.564578	0.387111	0.373316	0.400906	< .0001
probe14	cg09788843	154711081	0.401205	0.381329	0.421082	0.207031	0.194309	0.219753	< .0001
probe15	cg14435644	154711070	0.427333	0.401092	0.453573	0.140322	0.122054	0.15859	< .0001
probe16	cg07999845	154710961	0.608008	0.590549	0.625468	0.347042	0.329719	0.364366	< .0001
probe17	cg23910835	154710900	0.282087	0.264125	0.300005	0.112118	0.101651	0.122584	< .0001
probe18	cg23714408	154710873	0.412887	0.394964	0.43081	1.142367	0.135332	0.1494	< .0001
probe19	cg20881942	154710826	0.618749	0.597102	0.6404	0.205431	0.187188	0.223674	< .0001
probe20	cg00082664	154710796	0.557297	0.536445	0.57815	0.141551	0.124008	0.159094	< .0001
probe21	cg04965141	154710750	0.446823	0.422742	0.470904	0.067284	0.049592	0.08498	< .0001
probe22	cg25645268	154710598	0.509379	0.489361	0.529397	0.094929	0.077109	0.112749	< .0001
probe23	cg23207990	154710535	0.557931	0.542024	0.573837	0.215211	0.193531	0.236891	< .0001
	cg05961809	154710523		N/A			N/A		
probe24	cg22178613	154710499	0.564666	0.547876	0.581456	0.209478	0.188286	0.23033	< .0001
probe25	cg14289246	154710475	0.489134	0.467941	0.510327	0.070831	0.06363	0.07803	< .0001
probe26	cg10942078	154710429	0.550594	0.536523	0.564665	0.259156	0.246501	0.27181	< .0001
probe27	cg23292160	154710425	0.538231	0.523461	0.553	0.227442	0.212592	0.242293	< .0001
probe28	cg23121156	154710421	0.583444	0.567347	0.599542	0.230138	0.21076	0.249515	< .0001
probe29	cg05164933	154710418	0.632	0.615243	0.648756	0.212933	0.191674	0.234193	< .0001
probe30	cg14330641	154710399	0.470949	0.448926	0.492972	0.072687	0.067184	0.07819	< .0001
probe31	cg25775322	154710373	0.590979	0.573397	0.60856	0.201691	0.186707	0.216675	< .0001
probe32	cg11354906	154710371	0.541242	0.521375	0.56111	0.129538	0.11859	0.14049	< .0001
probe33	cg03202804	154710353	0.578239	0.556708	0.59977	0.110164	0.097535	0.122794	< .0001
probe34	cg06549216	154710224	0.368863	0.351034	0.386691	0.073178	0.067734	0.078622	< .0001
probe35	cg14063488	154709878	0.467953	0.444079	0.491826	0.059798	0.056423	0.063173	< .0001
probe36	cg05874561	154709828	0.493559	0.471156	0.515961	0.084422	0.076583	0.092261	< .0001
probe37	cg05774801	154709756	0.48362	0.463474	0.503767	0.147747	0.140021	0.155473	< .0001
probe38	cg07694025	154709441	0.398355	0.374705	0.422006	0.11367	0.099233	0.1235	< .0001
probe39	cg11467638	154707153	0.128191	0.123966	0.132416	0.122918	0.109557	0.136278	0.2253
probe40	cg24372829	154702806	0.887265	0.882836	0.891695	0.897804	0.89279	0.902819	0.1183
probe41	cg07859799	154702581	0.903236	0.895195	0.911277	0.932644	0.92959	0.935699	0.0158
probe42	cg10318528	154702461	0.312852	0.297616	0.328089	0.592453	0.567284	0.61762	< .0001

N/A denotes not available.

All *P* values was analyzed by analysis of variance.

Table S3. The comparison of the mean beta value between tumor and normal mucosa on *UNC5C* locus.

			UNC5C						
Probe name	Array ID	Genomic Coordinate	Tumor mean beta value			Normal beta value			*P* value
			Mean	Lower 95%CI	Upper 95%CI	Mean	Lower 95%CI	Upper 95%CI	
probe1	cg15701178	96471143	0.429316	0.410701	0.447932	0.061227	0.05174	0.070714	< .0001
probe2	cg12584684	96471105	0.584024	0.562114	0.60629	0.071318	0.064965	0.077671	< .0001
probe3	cg12802900	96470993	0.421782	0.407013	0.436552	0.145498	0.126571	0.164424	< .0001
probe4	cg21782409	96470887	0.563598	0.54399	0.583206	0.083342	0.062254	0.10443	< .0001
probe5	cg01194057	96470626	0.376197	0.357853	0.394541	0.04922	0.040258	0.058182	< .0001
probe6	cg13265789	96470584	0.615145	0.59711	0.633181	0.119727	0.102599	0.136855	< .0001
probe7	cg15984718	96470349	0.534644	0.522692	0.5466	0.214844	0.19271	0.23698	< .0001
probe8	cg17334018	96470293	0.45976	0.447372	0.472155	0.094711	0.074217	0.115205	< .0001
probe9	cg11723848	96470286	0.5109	0.49894	0.52286	0.121478	0.08603	0.15692	< .0001
probe10	cg22634891	96470250	0.405861	0.392051	0.419212	0.106556	0.089566	0.123545	< .0001
probe11	cg07234102	96470237	0.3102256	0.294884	0.325628	0.082049	0.071798	0.0923	< .0001
probe12	cg10520887	96470094	0.53037	0.51518	0.545557	0.231087	0.199063	0.263111	< .0001
probe13	cg00329039	96470053	0.413341	0.39768	0.429001	0.128753	0.113804	0.143703	< .0001
probe14	cg09100013	96469634	0.416798	0.39535	0.438245	0.086676	0.064563	0.108788	< .0001
probe15	cg16475705	96469565	0.356576	0.341743	0.371408	0.069864	0.057564	0.082165	< .0001
probe16	cg24870497	96469519	0.456713	0.438035	0.475391	0.056858	0.043408	0.070308	< .0001
probe17	cg23542495	96469343	0.472674	0.45471	0.490637	0.168513	0.13626	0.200763	< .0001
probe18	cg12412390	96469286	0.530793	0.51451	0.547079	0.2074	0.181225	0.233575	< .0001
probe19	cg01788994	96469264	0.238519	0.221294	0.255743	0.082296	0.06791	0.096677	< .0001
probe20	cg17031751	96469067	0.439172	0.417321	0.461023	0.110013	0.098006	0.122021	< .0001
probe21	cg07824265	96469029	0.272442	0.247847	0.297037	0.035627	0.03161	0.039646	< .0001
probe22	cg03008707	96468962	0.5898	0.574846	0.604753	0.238149	0.218449	0.257849	< .0001
	cg22490991	96465502		N/A			N/A		
probe23	cg02931225	96446177	0.609612	0.589114	0.63011	0.767302	0.730598	0.804006	< .0001
	cg14101976	96445972		N/A			N/A		
probe24	cg21574186	96445866	0.656029	0.635252	0.676805	0.857511	0.84743	0.867592	< .0001
	cg04281738	96445830		N/A			N/A		
probe25	cg05121379	96433143	0.717585	0.699203	0.735967	0.873842	0.86221	0.885474	< .0001
probe26	cg10528218	96361328	0.362742	0.35302	0.372461	0.378593	0.362978	0.394209	0.2876
probe27	cg11519903	96300982	0.884589	0.87704	0.892135	0.914209	0.908492	0.919926	< .0001
probe28	cg17504765	96300958	0.923801	0.919108	0.928495	0.940682	0.935139	0.946225	< .0001
	cg01462668	96292858		N/A			N/A		
probe29	cg12553181	96255377	0.765758	0.745963	0.78555	0.93496	0.930459	0.939461	< .0001
probe30	cg17337917	96102203	0.839269	0.830052	0.848486	0.872724	0.858082	0.887367	< .0001
probe31	cg03024478	96090330	0.387465	0.3720885	0.4028405	0.731114	0.703927	0.758353	< .0001

N/A denotes not available.

All *P* values was analyzed by analysis of variance.

Table S4. The associations between clinical characteristics and methylation status in each locus.

EFEMP1

Characteristics		Region 1, no. (%) Methylated	Region 1, no. (%) Unmethylated	*P* value	Region 2, no. (%) Methylated	Region 2, no. (%) Unmethylated	*P* value
	Total (n=949)	746 (78.6)	203 (21.4)		839 (84.5)	154 (15.5)	
Age	Median (Range)	68 (21-95)	63 (30-88)	< .0001#	68 (23-95)	63 (21-88)	< .0001#
Gender	Male (n=558)	435 (78.0)	123 (22.0)	0.56*	469 (84.1)	89 (16.0)	0.43*
	Female (n=391)	311 (79.5)	80 (20.5)		336 (85.9)	55 (14.1)	
Stage	I (n=230)	194 (84.4)	36 (15.7)	0.007*	204 (88.7)	26 (11.3)	0.01*
	II (n=263)	206 (78.3)	57 (51.7)		226 (85.9)	37 (14.1)	
	III (n=298)	236 (79.2)	62 (20.8)		254 (85.2)	44 (14.8)	
	IV (n=158)	110 (69.6)	48 (30.4)		121 (76.6)	37 (23.4)	
Tumor Location	Right (n=307)	275 (89.6)	32 (10.4)	< .0001*	282 (91.9)	25 (8.1)	< .0001*
	Left (n=642)	471 (73.4)	171 (26.6)		523 (81.5)	119 (18.5)	
Histology	Wel (n=275)	216 (78.6)	59 (21.5)	0.43*	234 (85.1)	41 (14.9)	0.36*
	mod (n=571)	444 (77.8)	127 (22.2)		479 (83.9)	92 (16.1)	
	poor or muc (n=103)	86 (83.5)	17 (16.5)		92 (11.4)	11 (10.7)	
BRAF/KRAS mutation	*BRAF* mutant (n=49)	47 (95.9)	2 (4.1)	< .0001*	47 (95.9)	2 (4.1)	< .0001*
	KRAS mutant (n=291)	263 (90.4)	28 (9.6)		272 (93.5)	19 (6.5)	
	wild type (n=609)	436 (71.6)	173 (28.4)		486 (79.8)	123 (20.2)	
MSI status	MSI (n=65)	64 (98.5)	1 (1.5)	< .0001*	61 (93.9)	4 (6.2)	0.036*
	Non-MSI (n=884)	682 (77.2)	202 (22.9)		744 (84.2)	140 (15.8)	
POLE mutation	Mutant (n=4)	4 (100)	0 (0)	0.3*	3 (75.0)	1 (25.0)	0.58*
	Wild type (n=945)	742 (78.5)	203 (21.5)		802 (84.9)	143 (15.1)	

SFRP2

Characteristics		Region 1, no. (%) Methylated	Region 1, no. (%) Unmethylated	*P* value	Region 2, no. (%) Methylated	Region 2, no. (%) Unmethylated	*P* value
	Total (n=949)	796 (83.9)	153 (16.1)		629 (66.3)	320 (33.7)	
Age	Median (Range)	68 (21-95)	66 (34-87)	0.2#	68 (21-95)	65 (24-92)	0.0001#
Gender	Male (n=558)	466 (83.5)	92 (16.5)	0.71*	362 (64.9)	196 (35.1)	0.27*
	Female (n=391)	330 (84.4)	61 (15.6)		267 (68.3)	124 (31.7)	
Stage	I (n=230)	195 (84.8)	35 (15.2)	0.43*	159 (69.1)	71 (30.9)	0.39*
	II (n=263)	220 (83.7)	43 (16.4)		176 (66.9)	87 (33.1)	
	III (n=298)	255 (85.6)	43 (14.4)		198 (66.4)	100 (33.6)	
	IV (n=158)	126 (79.8)	32 (20.3)		96 (60.8)	62 (39.2)	
Tumor Location	Right (n=307)	268 (87.3)	39 (12.7)	0.048*	227 (73.9)	80 (26.1)	0.006*
	Left (n=642)	528 (82.2)	114 (17.8)		402 (62.6)	240 (37.4)	
Histology	Wel (n=275)	235 (85.5)	40 (14.6)	0.56*	185 (67.3)	90 (32.7)	0.17*
	mod (n=571)	473 (82.8)	98 (17.2)		368 (64.5)	203 (35.6)	
	poor or muc (n=103)	88 (85.4)	15 (14.6)		76 (73.8)	27 (26.2)	
BRAF/KRAS mutation	*BRAF* mutant (n=49)	46 (93.9)	3 (6.1)	< .0001*	44 (89.8)	5 (10.2)	< .0001*
	KRAS mutant (n=291)	268 (92.1)	23 (7.9)		225 (77.3)	66 (22.7)	
	wild type (n=609)	482 (79.2)	127 (20.9)		360 (59.1)	249 (40.9)	
MSI status	MSI (n=65)	58 (89.2)	7 (10.8)	0.22*	51 (78.5)	14 (21.5)	0.03*
	Non-MSI (n=884)	738 (83.5)	146 (16.5)		578 (65.4)	306 (34.6)	
POLE mutation	Mutant (n=4)	4 (100)	0 (0)	0.38*	4 (100)	0 (0)	0.15*
	Wild type (n=945)	792 (83.8)	153 (16.2)		625 (66.1)	320 (33.9)	

UNC5C

Characteristics		Region 1, no. (%) Methylated	Region 1, no. (%) Unmethylated	*P* value	Region 2, no. (%) Methylated	Region 2, no. (%) Unmethylated	*P* value
	Total (n=949)	796 (80.2)	197 (19.8)		771 (77.6)	222 (22.4)	
Age	Median (Range)	68 (23-95)	65 (21-88)	< .0001#	68 (23-95)	65 (21-92)	0.0005#
Gender	Male (n=558)	446 (79.9)	112 (20.1)	0.26*	437 (75.7)	140 (24.3)	0.0894*
	Female (n=391)	324 (82.9)	67 (17.1)		334 (80.3)	82 (19.7)	
Stage	I (n=230)	194 (84.4)	36 (15.7)	0.21*	182 (79.1)	48 (20.9)	0.004*
	II (n=263)	216 (82.1)	47 (18.9)		208 (79.1)	55 (20.9)	
	III (n=298)	240 (80.5)	58 (19.5)		249 (83.6)	49 (16.4)	
	IV (n=158)	120 (76.0)	38 (24.1)		109 (69.0)	49 (31.0)	
Tumor Location	Right (n=307)	273 (88.9)	34 (11.1)	< .0001*	273 (88.9)	34 (11.1)	< .0001*
	Left (n=642)	497 (77.4)	145 (22.6)		475 (74.0)	167 (26.0)	
Histology	Wel (n=275)	228 (82.9)	47 (17.1)	0.46*	224 (81.5)	51 (18.6)	0.39*
	mod (n=571)	456 (79.9)	115 (20.1)		442 (77.4)	129 (22.6)	
	poor or muc (n=103)	86 (83.5)	17 (16.5)		82 (79.6)	21 (20.4)	
BRAF/KRAS mutation	*BRAF* mutant (n=49)	47 (95.9)	2 (4.1)	< .0001*	46 (93.9)	3 (6.1)	< .0001*
	KRAS mutant (n=291)	268 (92.1)	23 (7.9)		258 (88.7)	33 (11.3)	
	wild type (n=609)	455 (74.7)	154 (25.3)		444 (72.9)	165 (27.1)	
MSI status	MSI (n=65)	57 (87.7)	8 (12.3)	0.16*	56 (86.2)	9 (13.9)	0.14*
	Non-MSI (n=884)	713 (80.7)	171 (19.3)		692 (78.3)	192 (21.7)	
POLE mutation	Mutant (n=4)	4 (100)	0 (0)	0.33*	3 (75.0)	1 (25.0)	0.85*
	Wild type (n=945)	766 (81.1)	176 (18.9)		745 (78.8)	200 (21.2)	

#*P* values were calculated by analysis of variance.

**P* values were calculated by the chi-squared test.

Wel denotes well-differentiated adenocarcinoma.

Mod denotes moderately-differentiated adenocarcinoma.

Poor or muc denotes poorly-differentiated or mucinous adenocarcinoma.

Table S5. Clinical feature of spreading methylation in *EFEMP1*, *SFRP2*, and *UNC5C*

Characteristics		*EFEMP1*, no. (%)			*P* value	*SFRP2*, no. (%)			*P* value	*UNC5C*, no. (%)			*P* value
		Extensivly Methylated	Partially Methylated	Unmethylated		Extensivly Methylated	Partially Methylated	Unmethylated		Extensivly Methylated	Partially Methylated	Unmethylated	
	Total (n=949)	702 (74.0)	147 (15.5)	100 (10.5)		615 (64.8)	195 (20.6)	139 (14.7)		659 (69.4)	200 (21.1)	90 (9.5)	
Age	Median (Range)	68 (23-95)	64 (21-88)	63 (30-88)	< .0001#	68 (21-95)	66 (24-92)	65 (34-87)	0.0009#	68 (23-95)	66 (30-92)	61.5 (21-88)	< .0001#
Gender	Male (n=558)	407 (72.9)	90 (16.1)	61 (10.9)	0.69*	352 (63.1)	124 (22.2)	82 (14.7)	0.29*	373 (66.9)	131 (23.5)	54 (9.7)	0.08*
	Female (n=391)	295 (75.5)	57 (14.6)	39 (10.0)		263 (67.3)	71 (18.2)	57 (14.6)		286 (73.2)	69 (17.7)	36 (9.2)	
Stage	I (n=230)	185 (80.4)	28 (12.2)	17 (7.4)	0.003*	156 (67.8)	42 (18.3)	32 (13.9)	0.55*	167 (72.6)	42 (18.3)	21 (9.1)	0.08*
	II (n=263)	192 (73.0)	48 (18.3)	23 (8.8)		172 (65.4)	52 (19.8)	39 (14.8)		184 (70.0)	56 (21.3)	23 (8.8)	
	III (n=298)	222 (74.5)	46 (15.4)	30 (10.1)		196 (65.8)	61 (20.5)	41 (13.8)		215 (72.2)	59 (19.8)	24 (8.1)	
	IV (n=158)	103 (65.2)	25 (15.8)	30 (19.0)		91 (57.6)	40 (25.3)	27 (17.1)		93 (58.9)	43 (27.2)	22 (13.9)	
Tumor Location	Right (n=307)	267 (87.0)	23 (7.5)	17 (5.5)	< .0001*	226 (73.6)	43 (14.0)	38 (12.4)	0.0003*	250 (81.4)	46 (15.0)	11 (3.6)	< .0001*
	Left (n=642)	435 (67.8)	124 (19.3)	83 (12.9)		389 (73.6)	152 (23.7)	101 (15.7)		409 (63.7)	154 (24.0)	79 (12.3)	
Histology	Wel (n=275)	204 (74.2)	42 (15.3)	29 (10.6)	0.57*	183 (66.6)	54 (19.6)	38 (13.8)	0.12*	200 (72.7)	52 (18.9)	23 (8.4)	0.13*
	mod (n=571)	417 (73.0)	89 (15.6)	65 (11.4)		356 (62.4)	129 (22.6)	86 (15.1)		382 (66.9)	134 (23.5)	55 (9.6)	
	poor or muc (n=103)	81 (78.6)	16 (15.5)	6 (5.8)		76 (73.8)	12 (11.7)	15 (14.6)		77 (74.8)	14 (13.6)	12 (11.7)	
BRAF/KRAS mutation	*BRAF* mutant (n=49)	45 (91.8)	4 (8.2)	0 (0)	< .0001*	43 (87.8)	4 (8.2)	2 (4.1)	< .0001*	45 (91.8)	3 (6.1)	1 (2.0)	< .0001*
	KRAS mutant (n=291)	255 (87.6)	25 (8.6)	11 (3.8)		224 (77.0)	45 (15.5)	22 (7.6)		244 (83.9)	38 (13.1)	9 (3.1)	
	wild type (n=609)	402 (66.0)	118 (19.4)	89 (14.6)		348 (57.1)	146 (24.0)	115 (18.9)		370 (60.8)	159 (26.1)	80 (13.1)	
MSI status	MSI (n=65)	61 (93.9)	3 (4.6)	1 (1.5)	0.0007*	50 (76.9)	9 (13.9)	6 (9.2)	0.11*	51 (78.5)	11 (16.9)	3 (4.6)	0.21*
	Non-MSI (n=884)	641 (72.5)	144 (16.3)	99 (11.2)		565 (63.9)	186 (21.0)	133 (15.1)		608 (68.8)	189 (21.4)	87 (9.8)	
POLE mutation	Mutant (n=4)	3 (75.0)	1 (25.0)	0 (0)	0.72*	4 (100)	0 (0)	0 (0)	0.34*	3 (75.0)	1 (25.0)	0 (0)	0.81*
	Wild type (n=945)	699 (74.0)	146 (15.5)	100 (10.6)		611 (64.7)	195 (20.6)	139 (14.7)		656 (69.4)	199 (21.1)	90 (9.5)	

#*P* values were calculated by analysis of variance.
**P* values were calculated by the chi-squared test.
Wel denotes well-differentiated adenocarcinoma.
Mod denotes moderately-differentiated adenocarcinoma.
Poor or muc denotes poorly-differentiated or mucinous adenocarcinoma.

Table S6. Associations between clinical characteristics and the methylation score.

Characteristics		Mean methylation score (95%CI)	*P*-value*	Methylation Score, n (%)						
				0	1	2	3	4	5	6
Total (n=949)		4.7 (4.6-4.8)		22 (2.3)	33 (3.5)	61 (6.4)	82 (8.6)	115 (12.1)	183 (19.3)	453 (47.7)
Age	≥ 65 (n=580)	4.9 (4.8-5.1)	< .0001	9 (1.6)	15 (2.6)	26 (4.5)	42 (7.2)	63 (10.9)	120 (20.7)	305 (52.6)
	<65 (n=369)	4.3 (4.2-4.5)		13 (3.5)	18 (4.9)	35 (9.5)	40 (10.8)	52 (14.1)	63 (17.1)	148 (40.1)
Gender	Male (n=558)	4.7 (4.5-4.8)	0.12	14 (2.5)	22 (3.9)	36 (6.5)	47 (8.4)	75 (13.4)	110 (19.7)	254 (45.5)
	Female (n=391)	4.8 (4.7-5.0)		8 (2.1)	11 (2.8)	25 (6.4)	35 (9.0)	40 (10.2)	73 (18.7)	199 (50.9)
Stage	I (n=230)	4.9 (4.7-5.1)	0.018	9 (3.9)	3 (1.3)	10 (4.4)	15 (6.5)	28 (12.2)	42 (18.3)	123 (53.5)
	II (n=263)	4.8 (4.6-5.0)		4 (1.5)	14 (5.3)	12 (4.6)	20 (7.6)	29 (11.0)	66 (25.1)	118 (44.9)
	III (n=298)	4.8 (4.6-5.0)		2 (0.7)	6 (2.0)	23 (7.7)	30 (10.1)	40 (13.4)	52 (17.5)	145 (48.7)
	IV (n=158)	4.3 (4.0-4.6)		7 (4.4)	10 (6.3)	16 (10.1)	17 (10.8)	18 (11.4)	23 (14.6)	67 (42.4)
Tumor Location	Right (n=307)	5.2 (5.1-5.4)	< .0001	4 (1.3)	7 (2.3)	8 (2.6)	17 (5.5)	27 (8.8)	48 (15.6)	196 (63.8)
	Left (n=642)	4.5 (4.4-4.6)		18 (2.8)	26 (4.1)	53 (8.3)	65 (10.1)	88 (13.7)	135 (21.0)	257 (40.0)
Histology	Wel (n=275)	4.8 (4.6-5.0)	0.05	9 (3.3)	8 (2.9)	12 (4.4)	20 (7.3)	32 (11.6)	62 (22.6)	132 (48.0)
	mod (n=571)	4.7 (4.5-4.8)		11 (1.9)	22 (3.9)	42 (7.4)	55 (9.6)	73 (9.6)	109 (19.1)	259 (45.4)
	poor or muc (n=103)	5.0 (4.6-5.3)		2 (1.9)	3 (2.9)	7 (6.8)	7 (6.8)	10 (9.7)	12 (11.7)	62 (60.2)
BRAF/KRAS mutation	*BRAF* mutant (n=49)	5.7 (5.4-5.9)	< .0001	0 (0)	1 (2.0)	0 (0)	0 (0)	3 (6.1)	6 (12.2)	39 (79.6)
	KRAS mutant (n=291)	5.3 (5.2-5.5)		0 (0)	6 (2.1)	4 (1.4)	14 (4.8)	25 (8.6)	54 (18.6)	188 (64.6)
	Wild type (n=609)	4.4 (4.2-4.5)		22 (3.6)	26 (4.3)	57 (9.4)	68 (11.2)	87 (14.3)	123 (20.2)	226 (37.1)
MSI status	MSI (n=65)	5.3 (5.1-5.6)	0.001	0 (0)	0 (0)	2 (3.1)	5 (7.7)	5 (7.7)	10 (15.4)	43 (66.2)
	Non-MSI (n=884)	4.7 (4.6-4.8)		22 (2.5)	33 (3.7)	59 (6.7)	77 (8.7)	110 (12.4)	173 (19.6)	410 (46.4)
POLE mutation	Mutant (n=4)	5.5 (3.9-7.1)	0.31	0 (0)	0 (0)	0 (0)	0 (0)	1 (25.0)	0 (0)	3 (75.0)
	Wild type (n=945)	4.7 (4.6-4.8)		22 (2.3)	33 (3.5)	61 (6.4)	82 (8.6)	114 (12.1)	183 (19.4)	450 (47.6)

**P*-values were estimated by the Wilcoxon rank-sum test.

Wel denotes well-differentiated adenocarcinoma.

Mod denotes moderately-differentiated adenocarcinoma.

Poor or muc denotes poorly-differentiated or mucinous adenocarcinoma.

Table S7. Clinical characteristics of CRC patients and control subjects for ccfDNA methylation analyses.

Characteristics		Blood samples, n (%)							
		CRC Patient					Control Subject		
		Total (n=97)	pStage I (n=23)	pStage II (n=26)	pStage III (n=27)	pStageIV (n=21)	Total (n=62)	AP (n=16)	NN (n=46)
Age	≥ 65	75 (77.3)	19 (82.6)	21 (80.8)	19 (70.4)	16 (76.2)	33 (53.2)	10 (62.5)	23 (50.0)
	< 65	22 (22.7)	4 (17.4)	5 (19.2)	8 (29.6)	5 (23.8)	29 (46.8)	6 (37.5)	23 (50.0)
Gender	Male	42 (43.3)	9 (39.1)	12 (46.2)	11 (40.7)	10 (47.6)	36 (58.1)	8 (50.0)	28 (60.9)
	Female	55 (56.7)	14 (60.9)	14 (53.8)	16 (59.3)	11 (52.4)	26 (41.9)	8 (50.0)	18 (39.1)
CEA (ng/mL)	> 5.0	35 (36.1)	4 (17.4)	9 (34.6)	9 (33.3)	13 (61.9)	0 (0)	0 (0)	0 (0)
	≤ 5.0	62 (63.9)	19 (82.6)	17 (65.4)	18 (66.7)	8 (38.1)	62 (100)	16 (100)	46 (100)
cStage	I	21 (21.7)	13 (56.5)	3 (11.5)	4 (14.8)	1 (4.8)			
	II	29 (29.9)	9 (39.1)	15 (57.7)	5 (18.5)	0 (0)			
	III	30 (30.9)	1 (4.3)	8 (30.8)	18 (66.7)	3 (14.3)			
	IV	17 (17.5)	0 (0)	0 (0)	0 (0)	17 (81.0)			
Tumor Location	Right	33 (34.0)	7 (30.4)	11 (42.3)	9 (33.3)	6 (28.6)			
	Left	64 (66.0)	16 (69.6)	15 (57.7)	18 (66.7)	15 (71.4)			
Histology	Wel	23 (23.7)	10 (43.5)	9 (34.6)	4 (14.8)	0 (0)			
	Mod	65 (67.0)	12 (52.2)	13 (50.0)	22 (81.5)	18 (85.7)			
	Poor or muc	9 (9.3)	1 (4.3)	4 (15.4)	1 (3.7)	3 (14.3)			
ccfDNA concentration (ng/mL)	Mean (95%CI)	15.2 (13.5-16.9)	13.9 (11.9-15.9)	14.8 (12.2-17.4)	11.8 (10.0-13.7)	21.5 (15.2-27.7)	9.5 (8.7-10.3)	9.5 (8.0-11.0)	9.5 (8.5-10.5)

The pathological stage (pStage) is a classification based on pathological findings.
The clinical stage (cStage) is a classification based on pre-treatment clinical findings.
Control subjects categorized as adenomatous polyps (AP) were confirmed to have adenomatous polyps in the colon and rectum by colonoscopy.
Control subjects categorized as patients with no neoplasia (NN) were confirmed to have no adenomatous polyps in the colon and rectum by colonoscopy.

Table S8. The cut-off values of scores for sensitivity in patients with colorectal cancer (CRC) who are correctly identified as having the disease (true positives), as well as their 1-specificity for the proportion of individuals without the disease who are incorrectly identified as having the disease (false positives).

Score	Cut-off value	Sensitivity	1-Specificity	True postitive	True negative	False positive	False negative
	12	2.1%	0.0%	2	62	0	95
	10	3.1%	0.0%	3	62	0	94
	8	6.2%	0.0%	6	62	0	91
	7	9.3%	0.0%	9	62	0	88
	6	18.6%	0.0%	18	62	0	79
**The two-time methylation score**	5	30.0%	1.6%	29	61	1	68
	4	50.5%	8.1%	49	57	5	48
	3	70.1%	12.9%	68	54	8	29
	2	90.0%	40.3%	87	37	25	10
	1	100.0%	82.3%	97	11	51	0
	0	100.0%	100.0%	97	0	62	0
	12	90.7%	38.7%	88	38	24	9
	11	95.9%	58.1%	93	26	36	4
	10	97.9%	67.7%	95	20	42	2
**The two-time recovery score**	9	100.0%	79.0%	97	13	49	0
	8	100.0%	91.9%	97	5	57	0
	7	100.0%	95.2%	97	3	59	0
	6	100.0%	95.4%	97	1	61	0
	3	100.0%	100.0%	97	0	62	0
	24	2.1%	0.0%	2	62	0	95
	22	3.1%	0.0%	3	62	0	94
	20	6.2%	0.0%	6	62	0	91
	19	9.3%	0.0%	9	62	0	88
	18	17.5%	0.0%	17	62	0	80
	17	28.9%	1.6%	28	61	1	69
	16	48.5%	4.8%	47	59	3	50
	15	68.0%	8.1%	66	57	5	31
**The conbination score (Fc)**	14	86.6%	19.4%	84	50	12	13
	13	96.9%	41.9%	94	36	26	3
	12	97.9%	59.7%	95	25	37	2
	11	99.0%	71.0%	96	18	44	1
	10	100.0%	87.1%	97	8	54	0
	9	100.0%	90.3%	97	6	56	0
	8	100.0%	93.6%	97	4	58	0
	7	100.0%	98.4%	97	1	61	0
	3	100.0%	100.0%	97	0	62	0

Table S9. Clinical information and methylation status in the three genes of the six mCRC patients.

Patient No.	Methylation Ratio *EFEMP1*		*SFRP2*		*UNC5C*		Gender	Age (yrs)	Primary tumor Location	Histology	MSI status	RAS/BRAF mutational status	UICC Stage T	N	M	H	P	PUL	Clinical Stage at diagnosis
	Region 1	Region 2	Region 1	Region 2	Region 1	Region 2							T	N	M	H	P	PUL	Clinical Stage at diagnosis
1	0.56	0.78	0.78	0.66	0.71	0.72	Female	68	Transverse	Poor	MSI-High	*BRAF* V600E	3	1b	1a	0	1	0	IVB
2	0.90	0.91	0.89	0.88	0.97	0.86	Male	66	Rectosigmoid	Well	Non-MSI	Wild-type	3	1b	1a	2	0	0	IVA
3	0.01	0.00	0.32	0.25	0.51	0.17	Female	74	Sigmoid	Mod	Non-MSI	Wild-type	4b	0	1b	2	3	1	IVB
4	0.27	0.36	0.34	0.23	0.08	0.19	Male	60	Ascending	Poor	Non-MSI	*BRAF* V600E	2	2b	1a	0	0	0	IVA
5	0.00	0.43	0.62	0.79	0.73	0.00	Female	66	Sigmoid	Mod	Non-MSI	Wild-type	4a	0	1a	1	0	0	IVA
6	0.00	0.00	0.80	0.85	0.00	0.00	Male	40	Rectum	Mod	Non-MSI	Wild-type	3	0	1a	0	0	1	IVA

Table S10. Primer Sets of The Modified Combined Bisulfite Restriction Analysis (COBRA) with Fluorescence Dyes and High-Sensitive Assay for Bisulfite DNA (Hi-SA).

Locus		Primer	Temperature degrees Celsius (number of PCR)		Product size, base pair (restriction enzyme)
EFEMP1	Region 1	F; GAGCGAGTTTAGTATTTATTGGGT<br>R; ACCCTACAC AATCCAAAACAATTCT<br>IM; AGCGAGTTTAGTATTTATTGGGTC	60 (5), 58 (10), 56 (35)	ATTO565 (reverse) (Red)	98, 68*, 71* (HhaI)
	Region 2	F; TGTTGGGATGGGTATGTGTGT<br>R; CTAATCCCCTAAACTTTCAAAACCA<br>IM;TTGGGATGGGTATGTGTGTGC	60 (5), 58 (10), 56 (35)	ATTO550 (reverse) (Black)	86, 63*(HhaI)
SFRP2	Region 1	F; GGTYGGAGTTTTTYGGAGTTG<br>R: AACCCRCTCTCTTCRCTAAATAC<br>IM; **C**GGAGTTTTT**C**GGAGTTG**C**	60 (5), 58 (10), 56 (35)	ATTO550 (reverse) (Black)	140, 119*, 117*, 115*(Hha I)
	Region 2	F; GGTTGTTAGTTTTTYGGGGTTT<br>R; CAACRAACCAAAACCCTACAACAT<br>IM; **G**AACCAAAACCCTACAACATC**G**	60 (5), 58 (10), 56 (35)	ATTO565 (forward) (Red)	153, 121*(Hha I)
UNC5C	Region 1	F; GTYGGTTGGGGTAGTGTAAAAGTT<br>R; AACCTACCRCCTACCACCTTCTTC<br>IM; AACCTACCGCCTACCACCTTCTTCG	60 (5), 58 (10), 56 (35)	HEX (forward) (Green)	175, 146*(Hha I)
	Region 2	F; TTTAGTGGGGTTTTTAGTTGTTTG<br>R; TATCCCAATCCCAATCCRCAAC<br>IM; TATCCCAATCCCAATCCGCAACG	60 (5), 58 (10), 56 (35)	FAM (reverse) (Blue)	125, 100*(Hha I)

F: forward primer; R: reverse primer; IM: internal methylation-specific primer. The underlined nucleotides correspond to the methylated cytosines.
COBRA was done by F and R primers. Hi-SA was done by F, R, and IM primers.
*Length of cleaved methylated allele.
